# Recognizing autoimmunity in a breast cancer survivor with cutaneous fibrosis

**DOI:** 10.1016/j.jdcr.2026.01.011

**Published:** 2026-01-19

**Authors:** Kaela Williams, Meredith Park, Nicole Orzechowski, Margaret Coates

**Affiliations:** a University of North Carolina School of Medicine, Chapel Hill, North Carolina; b Department of Dermatology, University of North Carolina School of Medicine, Chapel Hill, North Carolina; c Division of Rheumatology, Department of Medicine, University of North Carolina School of Medicine, Chapel Hill, North Carolina

**Keywords:** limited cutaneous systemic sclerosis, radiation-induced morphea, systemic sclerosis

## Introduction

Radiation-induced morphea (RIM) is a rare complication of radiation therapy that presents as inflammatory fibrosis, most commonly in breast cancer patients.^1^ Only 0.2% of patients undergoing radiation for breast cancer develop RIM, which can delay diagnosis and treatment.^2^ Systemic sclerosis (SSc) is a complex autoimmune connective tissue disease characterized by progressive tissue and organ fibrosis.^3^ Early diagnosis of SSc is essential for slowing disease progression.^4^ Historically, diagnosis was difficult due to the heterogeneity of clinical presentation; however, criteria developed by the American College of Rheumatology and the European League Against Rheumatism in 2013 have provided a standardized approach to evaluating patients with symptoms concerning SSc. Here, we report on a patient with a history of breast cancer, initially diagnosed with RIM, who was later found to meet criteria for limited cutaneous systemic sclerosis (lcSSc).

### Case report

A 58-year-old woman with a dermatologic history of atopic dermatitis, Raynaud’s phenomenon, and scalp psoriasis was diagnosed in December 2022 with stage IIIB estrogen receptor-positive, progesterone receptor-negative, and human epidermal growth factor receptor 2–positive invasive ductal carcinoma of the right breast. Staging revealed ipsilateral axillary lymph node involvement without evidence of distant metastases.

Beginning in February 2023, the patient received neoadjuvant chemotherapy to the right side of the chest and supraclavicular region (28 fractions at 180 cGy), followed by bilateral skin-sparing mastectomies with right axillary lymphadenectomy and placement of 350 mL tissue expanders in July 2023. Adjuvant radiation therapy to the mastectomy scar (5 fractions at 100 cGy) was completed from August to September 2023, and targeted human epidermal growth factor receptor 2 therapy with trastuzumab-emtansine was administered from August 2023 through May 2024. Endocrine therapy with letrozole began in April 2024 but was self-discontinued in March 2025 due to the patient’s prioritization of scleroderma management.

The patient first noted right shoulder tightness and discomfort in October 2023. This was initially attributed to postradiation fibrosis and adhesive capsulitis. Following a motor vehicle accident in February 2024, her discomfort intensified with radiation into the neck and scapula, although imaging excluded acute injury. Despite consistent physical and occupational therapy, her right-shoulder tightness and upper-extremity discomfort progressed.

**Fig 1:**
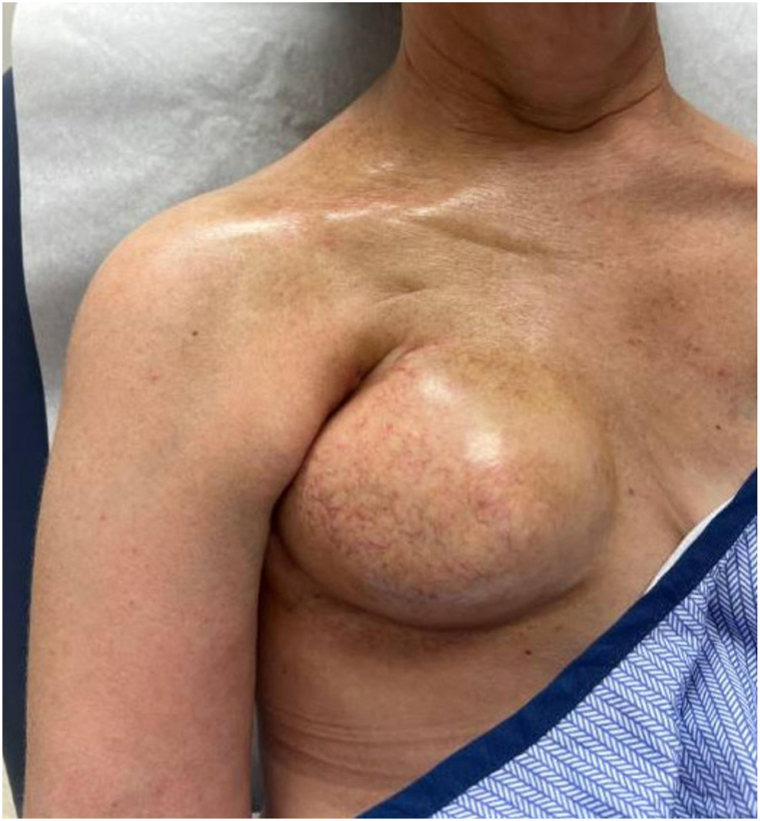
Dermatologic evaluation in March 2025 denoting significant and progressive fibrosis of the wall on the right side of the chest and right shoulder region, with distribution and severity suggestive of systemic fibrosing.

**Fig 2:**
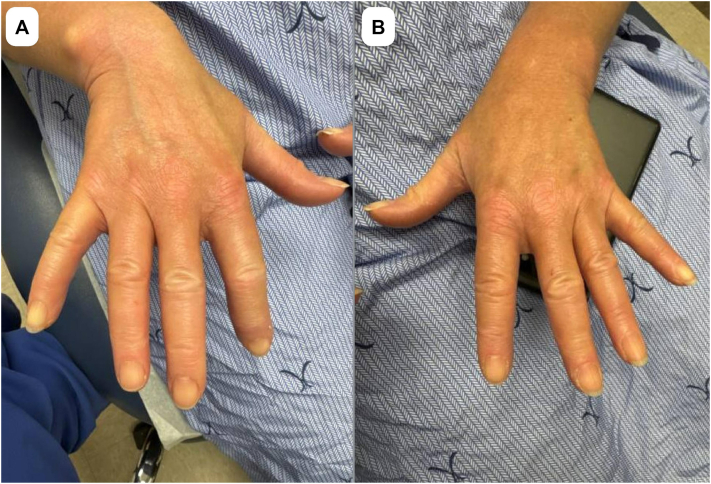
Dermatologic evaluation in March 2025 demonstrating puffy digits, dilated nailfold capillaries, and sclerodactyly consistent with limited cutaneous systemic sclerosis (lcSSc). Panel **(A)** shows the right hand, and panel **(B)** shows the left hand.

In September 2024, examination revealed marked capsular contracture of the right expander with overlying dermal thickening extending into the axilla and clavicle. A chest computed tomography scan in December 2024 demonstrated dense chest wall fibrosis and multiple right–sided sclerotic rib fractures without malignancy. These findings were out of proportion to expected postradiation effects, and a diagnosis of RIM was considered. Histologic changes consistent with morphea were seen on punch biopsy. The biopsy report revealed a mild inflammatory host response. The epidermis was atrophied with few dyskeratotic keratinocytes, whereas in the dermis, the collagen was thickened with hyalinization around adnexal units. Loss of adipose tissue and perivascular hyalinization were also seen. The patient was subsequently referred to dermatology, where evaluation in March 2025 showed significant fibrosis in the right side of the chest and right shoulder (Fig 1). She was also noted to have puffy digits, dilated nailfold capillaries, sclerodactyly, and facial telangiectasias at this visit, fulfilling the 2013 American College of Rheumatology/European League Against Rheumatism criteria for lcSSc (Fig 2). This diagnosis was further supported by a positive antinuclear antibody titer of 1:320 and RNA polymerase III antibody. Pulmonary function tests, creatine kinase, and echocardiography were unremarkable.

She was started on mycophenolate mofetil 1080 mg twice a day and a 3-month prednisone taper, beginning with 60 mg and ending with 30 mg. Initial topical treatment to the affected area included clobetasol 0.05% ointment twice a day before transitioning to steroid-sparing tacrolimus 0.1% ointment twice a day for maintenance therapy.

A comprehensive review of the patient’s records dating back to 2021 revealed early symptoms consistent with lcSSc, including esophageal dysmotility, Raynaud’s phenomenon, idiopathic peripheral neuropathy, unexplained hypertension, and an earlier high-titer antinuclear antibody (1:640). Unfortunately, further workup for these symptoms was not pursued at the time due to her subsequent breast cancer diagnosis.

## Discussion

Radiation-induced morphea represents localized fibrosis occurring in previously irradiated skin, typically within months to years following radiotherapy.^5^ The pathogenesis of RIM has been attributed to radiation-induced endothelial damage, immune dysregulation, and fibroblast overactivation, leading to excessive collagen deposition.^5^ Recent literature suggests that these fibrotic processes are more complex, with distinct fibroblast profiles that can extend outside the field of radiation.^6^ Myc overactivation has been identified as a major contributor to RIM development.^1^ In addition, RIM-derived fibroblasts have been found to express higher levels of osteopontin, a glycoprotein found downstream of the widely studied fibrotic mediator, transforming growth factor-beta.^1^ This case highlights the diagnostic challenge of distinguishing RIM from early SSc, particularly when sclerosis is confined to an irradiated field.

Early diagnosis of SSc is critical for mitigating disease progression and internal organ involvement, especially because specific therapies are indicated when pulmonary disease is present.^4^ This case demonstrates the importance of a comprehensive review of systems and physical examination when evaluating patients. Although initial histologic and radiologic findings of the right chest wall were consistent with RIM, a broader evaluation led to the correct diagnosis of lcSSc. Earlier identification and intervention could have reduced disease progression and improved the patient’s quality of life.

Few reports have described the development of SSc or RIM, and larger studies have shown conflicting evidence regarding the safety of radiotherapy in patients with preexisting SSc.^5^^,^^7^, ^8^, ^9^ SSc may occur as a paraneoplastic phenomenon and is associated with antibodies to RNA polymerase III, as was the case for our patient.^10^ It is possible that our patient developed lsSSc as a paraneoplastic phenomenon or had undiagnosed lcSSc at the time of radiation, given the presence of Raynaud’s phenomenon and esophageal dysmotility prior to her cancer diagnosis.

This case underscores the importance of early intervention and multidisciplinary collaboration, as expertise from dermatology, rheumatology, radiation oncology, and reconstructive surgery was instrumental in optimizing care. Our report illustrates the value of maintaining diagnostic vigilance and integrating longitudinal clinical information to minimize morbidity and improve outcomes in SSc.

## Conflicts of interest

None disclosed.
